# The Genome Copy Number of the Thermophilic Cyanobacterium Thermosynechococcus elongatus E542 Is Controlled by Growth Phase and Nutrient Availability

**DOI:** 10.1128/AEM.02993-20

**Published:** 2021-04-13

**Authors:** Sadaf Riaz, Meng Xiao, Pengyu Chen, Meijin Li, Yixuan Cui, Maurycy Daroch

**Affiliations:** a School of Environment and Energy, Peking University Shenzhen Graduate School, Shenzhen, China; North Carolina State University

**Keywords:** thermophile, cyanobacteria, *Thermosynechococcus*, polyploidy, genome copy number, ploidy

## Abstract

The recently isolated thermophilic cyanobacterium Thermosynechococcus elongatus PKUAC-SCTE542 (here *Thermosynechococcus* E542) is a promising strain for fundamental and applied research. Here, we used several improved ploidy estimation approaches, which include quantitative PCR (qPCR), spectrofluorometry, and flow cytometry, to precisely determine the ploidy level in *Thermosynechococcus* E542 across different growth stages and nutritional and stress conditions. The distribution of genome copies per cell among the populations of *Thermosynechococcus* E542 was also analyzed. The strain tends to maintain 3 or 4 genome copies per cell in lag phase, early growth phase, or stationary phase under standard conditions. Increased ploidy (5.5 ± 0.3) was observed in exponential phase; hence, the ploidy level is growth phase regulated. Nearly no monoploid cells were detected in all growth phases, and prolonged stationary phase could not yield ploidy levels lower than 3 under standard conditions. During the late growth phase, a significantly higher ploidy level was observed in the presence of bicarbonate (7.6 ± 0.7) and high phosphate (6.9 ± 0.2) at the expense of reduced percentages of di- and triploid cells. Meanwhile, the reduction in phosphates decreased the average ploidy level by increasing the percentages of mono- and diploid cells. In contrast, temperature and antibiotic stresses reduced the percentages of mono-, di-, and triploid cells yet maintained average ploidy. The results indicate a possible causality between growth rate, stress, and genome copy number across the conditions tested, but the exact mechanism is yet to be elucidated. Furthermore, the spectrofluorometric approach presented here is a quick and straightforward ploidy estimation method with reasonable accuracy.

**IMPORTANCE** The present study revealed that the genome copy number (ploidy) status in the thermophilic cyanobacterium *Thermosynechococcus* E542 is regulated by growth phase and various environmental parameters to give us a window into understanding the role of polyploidy. An increased ploidy level is found to be associated with higher metabolic activity and increased vigor by acting as backup genetic information to compensate for damage to the other chromosomal copies. Several improved ploidy estimation approaches that may upgrade the ploidy estimation procedure for cyanobacteria in the future are presented in this work. Furthermore, the new spectrofluorometric method presented here is a rapid and straightforward method of ploidy estimation with reasonable accuracy compared to other laborious methods.

## Abstract (precis)

The present study revealed that the genome copy number (ploidy) status in the thermophilic cyanobacterium *Thermosynechococcus* E542 is regulated by growth phase and various environmental parameters to give us a window into understanding the role of polyploidy. An increased ploidy level is found to be associated with higher metabolic activity and increased vigor by acting as backup genetic information to compensate for damage to the other chromosomal copies.

## INTRODUCTION

Cyanobacteria are very diverse and widely distributed photoautotrophic prokaryotes capable of converting light energy into chemical energy by oxygenic photosynthesis. Their simple nutritional requirements, complex photosynthetic system (1), high energy utilization rate (2), clear genetic background, and easy genetic manipulations (3) have spurred interest in using cyanobacterial cell factories to produce a variety of biochemicals. Thermophilic cyanobacteria thrive in high-temperature environments and are often more resistant to extreme conditions than mesophilic cyanobacteria (4). The thermophilic cyanobacterium Thermosynechococcus elongatus PKUAC-SCTE542 (here *Thermosynechococcus* E542) (5) is a promising strain for fundamental and applied research. The genome of *Thermosynechococcus* E542 consists of a circular genome (2.6 Mbp) without any plasmid, and it is naturally transformable (6).

Bacteria are very diverse concerning their genome copy numbers (ploidy). Many bacterial model strains such as Escherichia coli and Bacillus subtilis contain a single circular chromosome per cell (monoploidy) during slower growth (7, 8). Others, like Caulobacter crescentus, are monoploid irrespective of the growth rate (9). Alternatively, some bacteria maintain multiple chromosome copies per cell (polyploidy) irrespective of the growth rate, as observed in many cyanobacteria (10–13). Ploidy of cyanobacteria is highly variable; it is influenced by growth phase, chemical and physical parameters, and stress conditions (10, 12, 14, 15). Previous studies have discussed the various benefits of polyploidy in prokaryotes (16), including the low mutation rate, resistance against double-strand breaks, gene redundancy, global regulation of gene dosage, large cell size, and storage of phosphate. However, polyploid cells impede the construction of mutants, and segregating desired mutations across all genome copies is time-consuming, often taking more than a month (12).

Thermophilic cyanobacteria of the family *Thermosynechococcaceae*, including the recently isolated Thermosynechococcus elongatus E542 (FACHB-2455), are underexplored for their genetics compared to cyanobacterial model strains. To date, no ploidy level studies have been conducted for thermophilic cyanobacteria. The present study was intended to determine the ploidy level variations during different growth stages and nutritional and stress conditions to shed light on the regulation of DNA replication in cyanobacteria. We have employed three different ploidy estimation approaches, i.e., quantitative PCR (qPCR), spectrofluorometry, and flow cytometry, to precisely analyze the overall ploidy level per cell and the distribution of genome copies among *Thermosynechococcus* E542 populations. Our results reveal that ploidy in *Thermosynechococcus* E542 is growth phase regulated and influenced by nutrient availability. Furthermore, the results suggest that there is a correlation between increased growth and genome copy number. The direction of the cause and effect remains to be determined. In principle, two alternatives are possible: multiple copies of chromosomes in *Thermosynechococcus* E542 facilitate increased growth, or alternatively, the cell division rate does not match the DNA synthesis rate, resulting in increased ploidy. In summary, this study has explored the genome copy number variations in *Thermosynechococcus* E542 and discussed their alleged role across the conditions tested.

## RESULTS

### Optimization of qPCR and spectrofluorometric methods.

Precise measurement of genome copies using qPCR or a spectrofluorometer is mostly dependent on the accuracy of cell counts and cell lysis efficiency. We used an automated cell counting system rather than a traditional cell counting method. The correlation between the optical density (OD) and the cell count was above 99% (see Fig. S1 in the supplemental material). Furthermore, ploidy levels estimated by a cell count-dependent method (e.g., qPCR) and a cell count-independent method (flow cytometry) were in excellent agreement, suggesting considerable accuracy of the automated cell counter. It was also noted that a test sample cell density ranging between OD at 730 nm (OD_730_) values of 0.05 and 0.1 gives a more accurate cell count on the automated cell counter. The increased cell density resulted in a lower cell count due to higher cell aggregation.

Previous studies have tested different cell lysis methods for cyanobacterial cells, and cell lysis by a bead beater was found to be optimal for ploidy estimation (11). For cell lysis using the bead beater, glass and zirconium beads were tested. One-millimeter and 0.1-mm glass beads were found to be inefficient for *Thermosynechococcus* E542 cell lysis. The 0.1-mm zirconium beads give a satisfactory result of low DNA damage and high lysis efficiency. To determine the cell lysis efficiency, cells were mixed with 0.75 g of 0.1-mm zirconium beads and lysed on the bead beater for 60, 90, and 120 s. The results are summarized in Table S1 in the supplemental material.

An acceptable qPCR efficiency was obtained after a standard curve was constructed with the *Thermosynechococcus* E542 standard fragment and cell lysate (Fig. S2). Previous studies have used spectroscopic methods for ploidy estimation either after extracting DNA (17) or after removing RNA from the cell lysate (10). These methods may cause the loss of DNA during the precipitation stage and are time-consuming. We have employed a sensitive DNA binding fluorescent dye, and fluorescence was measured by a spectrofluorometer from the cell lysate directly. Genome copies obtained as a result of qPCR and spectrofluorometer measurements were comparable (Table S1).

### No-phosphate cells as a diploid control for flow cytometry.

**FIG 1:**
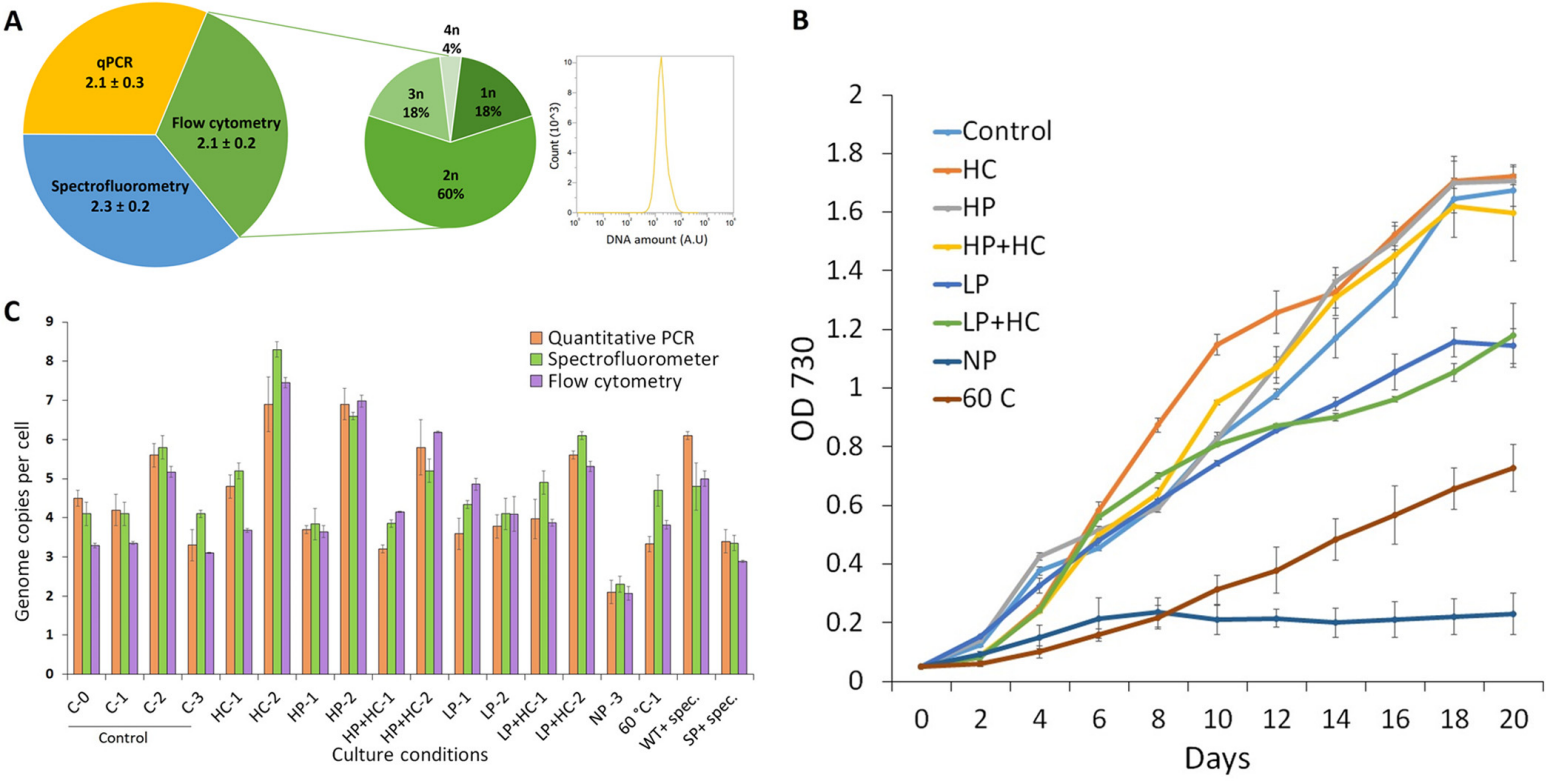
Growth curve and ploidy estimation of *Thermosynechococcus* E542. (A) Comparison of ploidy estimation of no-phosphate (NP) *Thermosynechococcus* E542 using different detection methods: qPCR (yellow), spectrofluorimetry (blue), and flow cytometry (green). The secondary pie chart shows the distribution of *Thermosynechococcus* E542 NP culture ploidy using the flow cytometry method and its corresponding histogram of DNA levels. A.U, arbitrary units. (B) Growth curve of *Thermosynechococcus* E542 grown under different nutritional conditions. Control represents cells grown in conventional BG-11. Other conditions are different nutrient variations of BG-11 according to Table 1. (C) Ploidy estimation under different test conditions using NP *Thermosynechococcus* E542 as the diploid control in flow cytometry. Three different detection methods are compared; results are shown as the averages from three biological replicates, and error bars show standard deviations. C, control; 0, lag phase; 1, early growth phase; 2, late growth phase; 3, stationary phase.

Previous studies have used Escherichia coli cells as a control for flow cytometry to determine the genome copy number in Synechococcus elongatus PCC 7942 (14) and *Synechocystis* sp. strain PCC 6803 (12). The genome of E. coli is 4.6 Mbp (7), while the genome of *Thermosynechococcus* E542 is 2.6 Mbp (6), which is 57% of the size of the E. coli genome. It was concluded that a different control would be more appropriate. In the present work, no-phosphate (NP) *Thermosynechococcus* E542 was found to have an average of 2.2 ± 0.1 genome copies per cell using qPCR and spectrofluorometric methods (Fig. 1A). The distribution of genome copies in the populations of NP *Thermosynechococcus* E542 and the related DNA histogram are shown in Fig. 1A. We used NP *Thermosynechococcus* E542 as a control to determine the average ploidy level in S. elongatus PCC 7942 (genome size, 2.6 Mbp). Our results (average ploidy, 3.1 ± 0.3) were comparable to those of previous studies (average ploidy, 3.3 ± 0.9 [14] and 4.0 ± 0.3 [11]). Fluorescence intensity readings and their corresponding genome copy numbers are provided in Table S2 in the supplemental material.

### Ploidy of *Thermosynechococcus* E542 at different growth stages in conventional BG-11.

In conventional BG-11, the specific growth rate of *Thermosynechococcus* E542 was recorded as 0.095 ± 0.008 day^−1^. The growth curve and estimated ploidy under each condition are shown in Fig. 1. Genome copies of *Thermosynechococcus* E542 were relatively stable at between 3.5 ± 0.5 and 4.0 ± 0.6 copies per cell during lag phase (OD_730_ of 0.08), early growth phase (OD_730_ of 0.3), and stationary phase (OD_730_ of 1.5) (*P* > 0.05 by a *t* test). In high-density culture, which is termed the late growth phase (OD_730_ of 0.9), the recorded ploidy was significantly higher (5.5 ± 0.3) than in the other growth stages (*P* < 0.05 by a *t* test) (Fig. 1C). Prolonged incubation of the stationary-phase culture (OD_730_ of 2.5) could not yield a ploidy level lower than 3 under standard conditions.

### Ploidy of *Thermosynechococcus* E542 under different nutritional conditions.

The addition of bicarbonates is reported to increase the growth of cyanobacteria (18), including thermophilic cyanobacteria (19). An increased specific growth rate (0.149 ± 0.005 day^−1^) was recorded in high-carbon (HC) medium. In the early growth phase of HC cultures, the growth rate decreased. However, a sudden increase in growth was observed after 4 days (OD_730_ of 0.4), and this increase remained dominant over other cultures until the HC culture reached an OD_730_ of 1.2 (Fig. 1B). Similar ploidy (4.6 ± 0.8) was observed at the early growth phase compared to the control (4.0 ± 0.6). However, significantly increased (*P* < 0.05 by a *t* test) 7.6 ± 0.7 genome copies per cell were found in late-growth-phase cultures, which is the highest genome copy number compared to the other tested conditions (Fig. 1C).

The specific growth rates of *Thermosynechococcus* E542 were recorded as 0.098 ± 0.003 day^−1^, 0.098 ± 0.001 day^−1^, 0.066 ± 0.001 day^−1^, and 0.060 ± 0.001 day^−1^ in HP (high-phosphate), HP plus HC (HP+HC), LP (low-phosphate), and LP+HC cultures, respectively. These results show that supplementation of carbonates does not influence the growth rate if the phosphate concentration diverges significantly from the 0.23 mM present in standard BG-11. In early-growth-phase cultures, comparable ploidy levels with no significant difference (*P* > 0.05 by a *t* test), 3.7 ± 0.1, 3.7 ± 0.5, 4.3 ± 0.6, and 4.3 ± 0.6, were observed in HP, HP+HC, LP, and LP+HC cultures, respectively (Fig. 1C). Variation in carbonate and phosphate concentrations does not influence the ploidy level in *Thermosynechococcus* E542 during the early growth phase. In late-growth-phase cultures, 6.9 ± 0.2, 5.7 ± 0.5, 4.0 ± 0.2, and 5.7 ± 0.4 genome copies per cell were recorded in HP, HP+HC, LP, and LP+HC cultures, respectively (Fig. 1C). During the late growth phase, an increase or decrease in the phosphate level significantly affected the ploidy level (*P* < 0.05 by a *t* test) compared to conventional BG-11 medium. DOE (design of experiments) factor analysis revealed the growth phase as the most significant factor (*P* value of 0.000), followed by the carbonate level (*P* value of 0.007), while the phosphate level was attributed as the least significant factor (*P* value of 0.083) influencing the ploidy level in *Thermosynechococcus* E542.

### Ploidy of *Thermosynechococcus* E542 under stress conditions.

The rise in temperature from 45°C to 60°C reduces the growth rate and biomass productivity in *Thermosynechococcus* E542 (6). Meanwhile, no change in the overall ploidy level (*P* > 0.05 by a *t* test) was observed between cells grown at 45°C (ploidy, 4.0 ± 0.6) and those grown at 60°C (ploidy, 4.0 ± 0.7) (Fig. 1C).

Figure S3 in the supplemental material shows the spectinomycin sensitivity of wild-type (WT) *Thermosynechococcus* E542 and Synechococcus elongatus PCC 7942. An increase in the ploidy level of 5.3 ± 0.7 was observed in WT *Thermosynechococcus* E542 at 2.5 μg/ml of spectinomycin. However, no significant difference (*P* > 0.05 by a *t* test) was found compared to the control. Transformed *Thermosynechococcus* E542 showed reduced ploidy (3.2 ± 0.3) at 50 μg/ml of spectinomycin, as opposed to the WT plus the spectinomycin control (*P* < 0.05 by a *t* test) (Fig. 1C).

### Distribution of genome copy numbers in populations of Thermosynechococcus elongatus E542.

Stress conditions such as nutritional starvation, high temperatures, or antibiotics might cause ploidy changes in cyanobacteria; these changes, however, were not very evident after the determination of average ploidy (*P* > 0.05 by a *t* test). Therefore, flow cytometry was employed to determine the distribution of genome copy numbers in populations of Thermosynechococcus elongatus E542.

**FIG 2:**
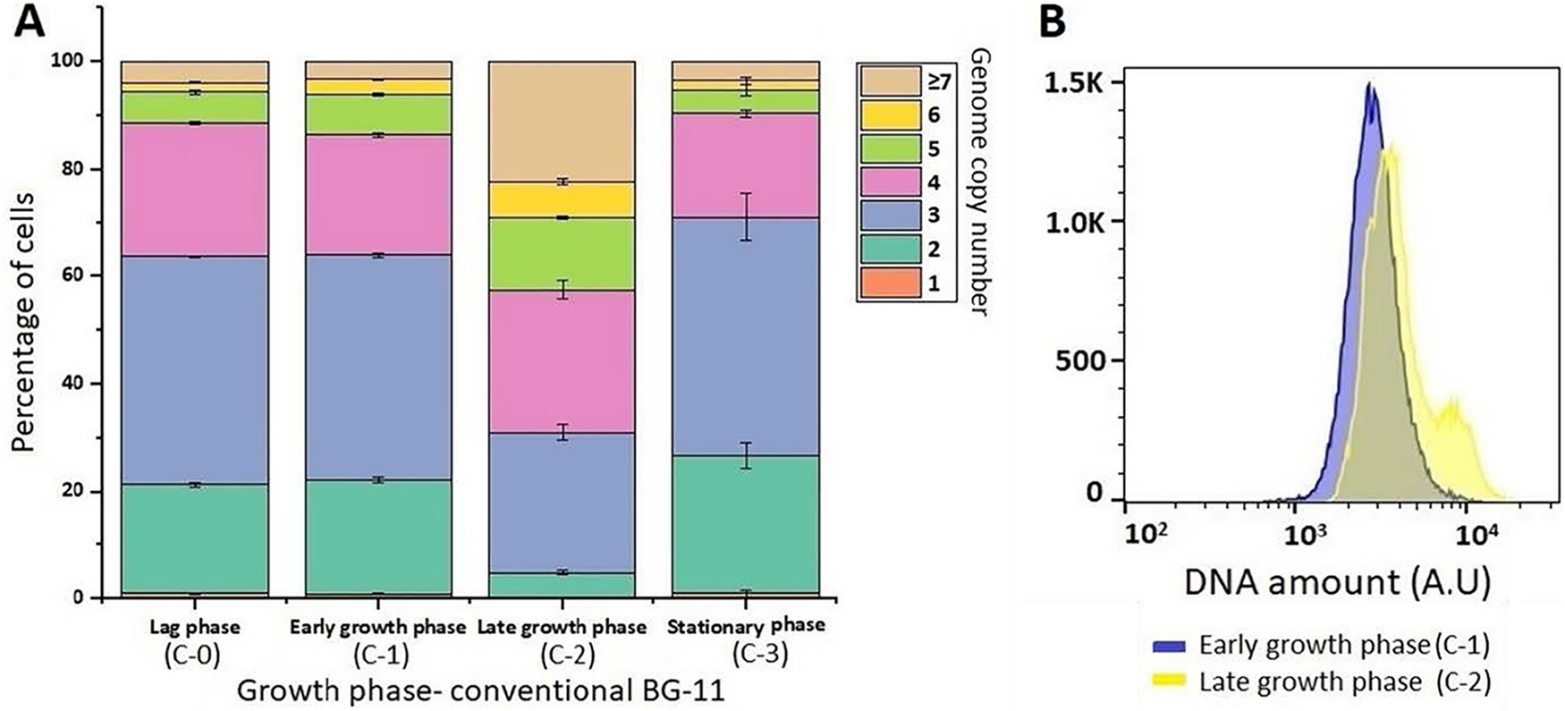
Determination of genome copies in Thermosynechococcus elongatus E542 at different growth stages in conventional BG-11. (A) Distribution of genome copy numbers in populations of *Thermosynechococcus* E542. (B) Flow cytometry analysis showing the DNA levels during early and late growth phases in conventional BG-11. A.U, arbitrary units.

In conventional BG-11, monoploid cells were nearly negligible. Lag-phase and early-growth-phase cultures displayed nearly the same distribution of genome copies, with 20% of cells possessing 2 genomes, while most of the cells (40%) displayed 3 genome copies (Fig. 2A). Nearly the same pattern is observed in stationary-phase cultures. Late-growth-phase cultures possessed only 4.86% ± 0.33% of the population with 2 genome copies. Cells with 3 genome copies were also reduced from 41.66% ± 0.41% to 26.23% ± 1.51%, while cells with more than 5 genome copies were observed in late-growth-phase cultures, making the total ploidy higher than 5. A DNA histogram also showed a shift in the DNA amount in early- and late-growth-phase cultures (Fig. 2B).

**FIG 3:**
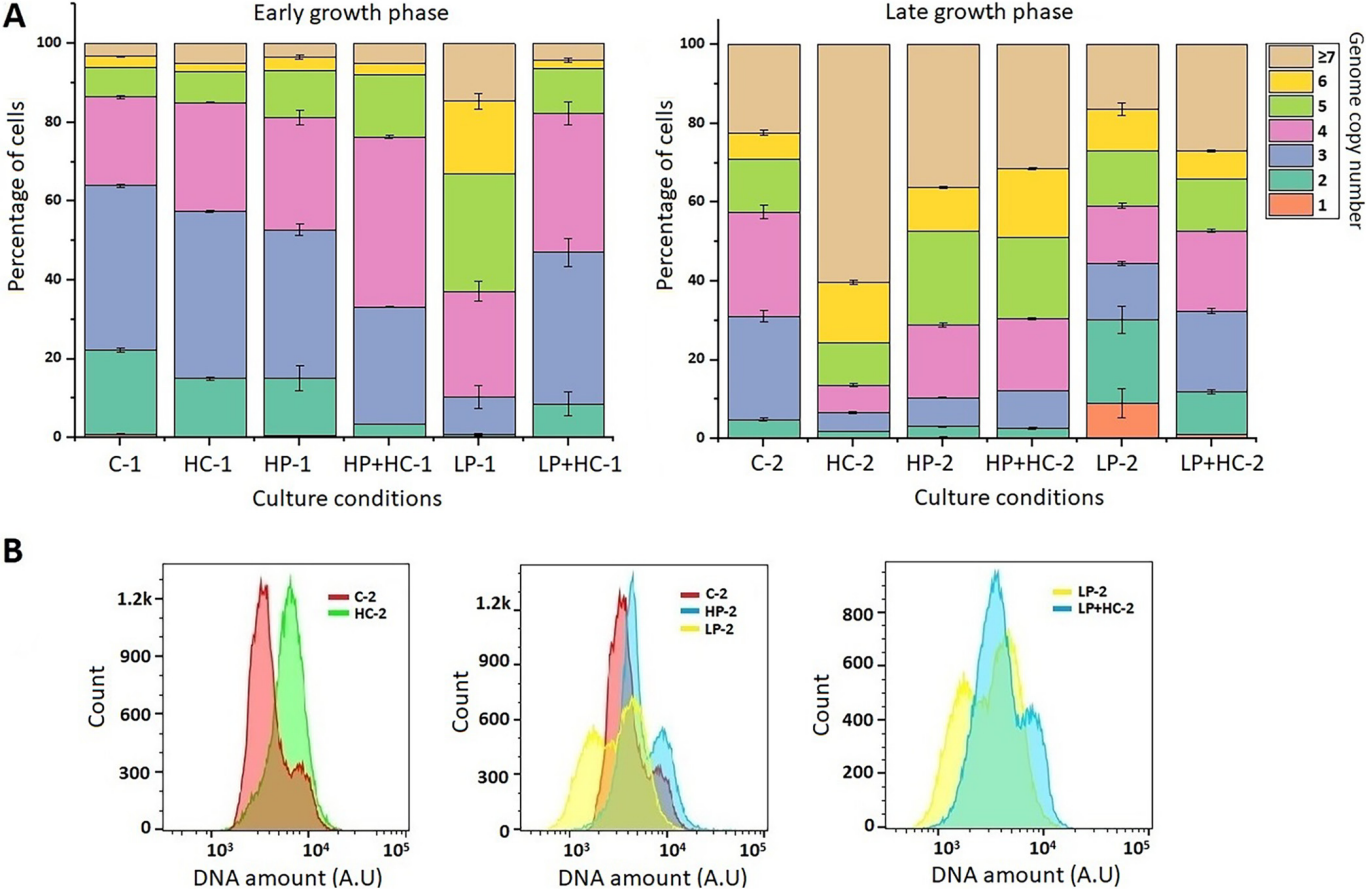
Determination of genome copies in *Thermosynechococcus* E542 under different nutritional conditions. (A) Distribution of genome copy numbers in populations of *Thermosynechococcus* E542. C, conventional BG-11; HC, high carbonate; HP, high phosphate; HP+HC, high phosphate with high carbonate; LP, low phosphate; LP+HC, low phosphate with high carbonate; 1, early growth phase; 2, late growth phase. (B) Flow cytometry analysis showing the DNA levels during late growth phases. A.U, arbitrary units.

At the early growth phase, under different nutritional conditions, a negligible number of monoploid cells was observed (Fig. 3A). *Thermosynechococcus* E542 control cells contained the highest percentage of cells (41.66% ± 0.41%) with 3 genome copies and showed a similar percentage (22.53% ± 0.41%) of cells containing 2 and 4 genome copies per cell. HC and HP cultures possessed slightly lower percentages of diploid cells (15%) than the control, and the highest percentages of populations were found to contain 3 genome copies (42% in HC and 37% in HP cultures). Interestingly, HP and HP+HC showed similar ploidy levels in early cultures. However, the distribution of genome copies among the population was different; e.g., HP+HC cultures possessed a reduced percentage of diploid cells (14% in HP and 3% in HP+HC cultures), with the highest percentage of cells with 4 genome copies (43%), which was 28% in HP cultures. Early LP cultures showed the highest percentage of cells (30%) containing 5 genome copies, higher than that under any other condition.

In the late growth phase, the cultures supplemented with carbonates displayed a lower number of cells with lower genome copy numbers, and a higher percentage of cells was observed to possess high genome copy numbers per cell. For the first time, the highest number of monoploid cells (9.05% ± 3.71%) was observed in LP cultures, compared to the other nutritional conditions. Although the LP cells displayed high levels of monoploid and diploid cells (21.06% ± 3.44%), it is also interesting to note that when LP medium was supplemented with carbonates, the numbers of monoploid (1.16% ± 0.05%) and diploid (10.76% ± 0.47%) cells were reduced with respect to LP medium (Fig. 3A). DNA histograms represent the increase in the DNA amount after the addition of carbonates to conventional BG-11 or LP medium (Fig. 3B). They also show an increase in the DNA amount with increased phosphate availability. Conversely, a decrease in the DNA amount is displayed by cultures with low phosphate levels.

**FIG 4:**
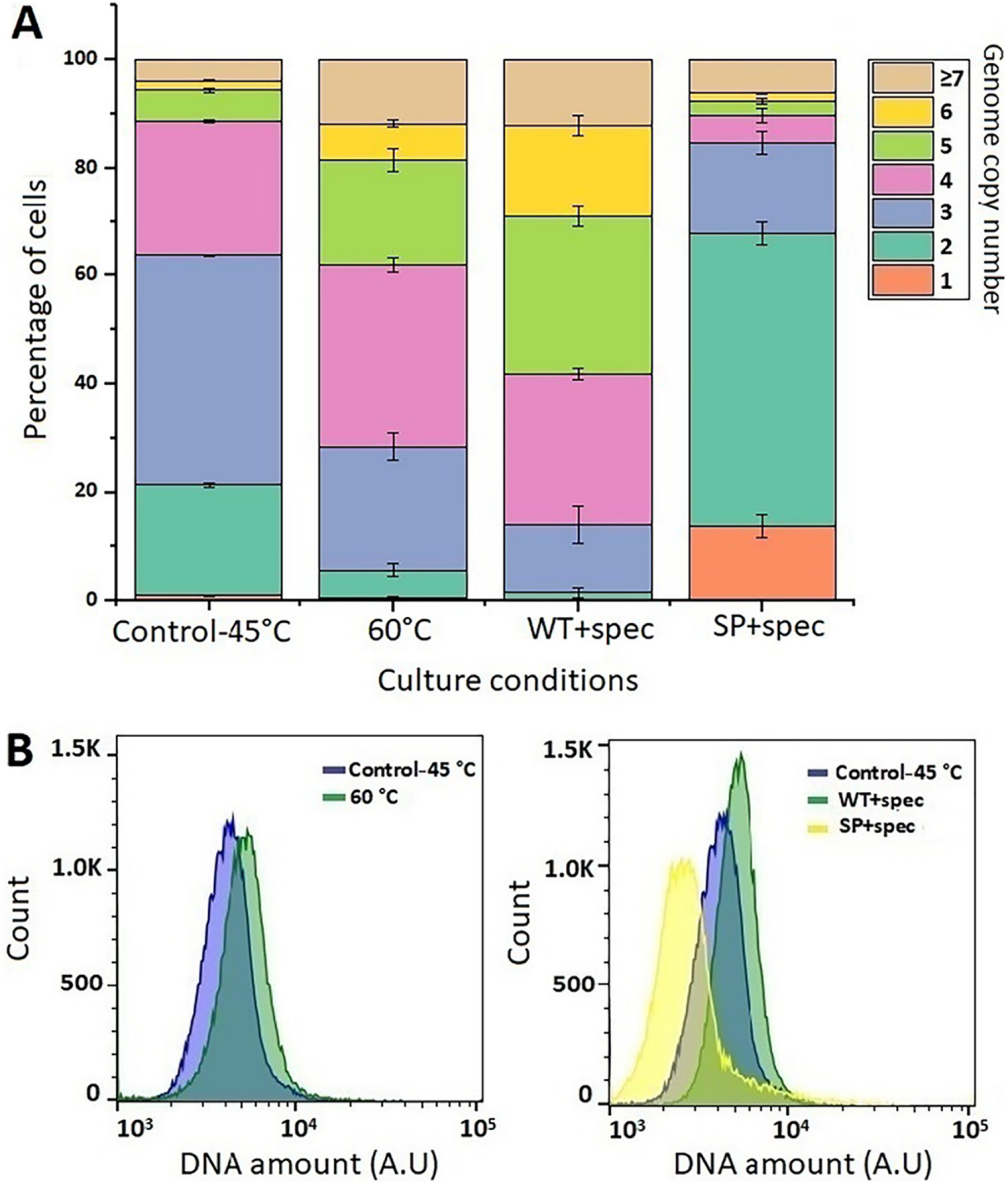
Determination of genome copies in *Thermosynechococcus* E542 under different stress conditions. (A) Distribution of genome copy numbers in populations of *Thermosynechococcus* E542. (Top) 45°C and 60°C; (bottom) control. WT+spec, wild-type cells in 2.5 μg of spectinomycin; SP+spec, transformed cells containing a spectinomycin-resistant plasmid grown in 50 μg of spectinomycin. (B) Flow cytometry analysis showing the DNA levels under temperature and antibiotic stress. A.U, arbitrary units.

Under different culture temperatures, cells grown at 45°C showed nearly 21% diploid cells, and the highest percentage of the population (40%) showed 3 genome copies (Fig. 4A). On the other hand, populations containing 2 and 3 genome copies were reduced from 21% to 5% or 41% to 23% at 60°C. The highest percentage of the population (33.53% ± 1.25%) contained 4 genome copies, while more cells possessing 5 and 6 genome copies were recorded at 60°C. DNA histograms represent the increase in the DNA amount at 60°C (Fig. 4B).

When BG-11 was supplemented with spectinomycin, WT *Thermosynechococcus* E542 had negligible monoploid and diploid cells, and the highest percentages of the population were found to contain 4 genomes (27.9% ± 1.05%), 5 genomes (28.86% ± 1.88%), and 6 genomes (17.06% ± 1.90%) per cell (Fig. 4A). In contrast, transformed cells showed that the highest percentage (53.96% ± 2.06%) of the population was diploid. DNA histograms represent the increase in the DNA amount when BG-11 was supplemented with spectinomycin for WT cells, while transformed cells showed a decrease in the DNA amount (Fig. 4B).

## DISCUSSION

### Comparison of different methods for ploidy estimation.

Several techniques are being used to detect the ploidy level. Methods include measurements of gene copies and enzyme activity, quantification of total DNA in a cell, and bioinformatics approaches (20, 21). Recent studies are improving these methods to make them more effective, reliable, and fast. The qPCR approach is considered fast and reliable (22). The qPCR method requires accurate cell numbers, maximum lysed cells, and the smallest amount of degraded genomic DNA (gDNA), quantified by qPCR. The ploidy level by qPCR could vary slightly with a different set of primers because of the different PCR amplification efficiencies. qPCR results depend on the selection of the genomic site being analyzed; for example, reduced ploidy was observed when a site near the terminus was analyzed compared to the site near the replication origin (23). This is likely due to the presence of more origins of replication (6.8) than its termini (2) in a fast-growing E. coli strain. Consequently, the time to replicate and segregate the chromosome becomes shorter than the cell doubling time. Since E. coli starts a new round of replication before the previous round has been terminated, a higher gene dosage of regions near the replication origin than those near the terminus occurs (23). Slightly lower ploidy was detected in the cyanobacterium *Synechocystis* PCC 6803 when a genome site near the terminus was selected for qPCR (10). However, DNA replication in *Synechocystis* does not occur at a defined origin (24), and it is still unknown how the process is controlled in *Thermosynechococcus* E542 or other *Thermosynechococcaceae*. As the PCR product size is relatively small in the qPCR approach (100 to 200 bp), a slight breakdown of DNA during cell lysis might not affect the overall results, and the cell lysate could be directly used as the template, whereas an accurate cell counting method remains crucial to achieve valid results. The major drawback of this approach is that it does not provide any information about the distribution of genome copies among a population.

The spectroscopic approach uses the measurement of the absorbance to quantify the total DNA amount in a sample. The spectrofluorometric approach is also dependent on a precise cell count and lysis method and an effective DNA extraction protocol. We have improved this method by using automated cell counting and DNA measurement directly from the cell lysate by using a sensitive DNA binding fluorescent dye. The spectrofluorometric method presented in this study is a quick and straightforward method of ploidy estimation with reasonable accuracy compared to other methods used in this work. Similar to qPCR, this approach could not detect the heterogeneity of genome copy numbers in a sample.

Although *Thermosynechococcus* E542 maintains relatively stable ploidy, it can also alter the genome copy number per cell in a population. Flow cytometry of DNA-stained cells was performed for the ploidy estimation. The added benefit of this approach is that it could determine the distribution of genome copies among the populations. However, sample storage, unknown parameters that interfere with DNA staining, and the lack of universally agreed-upon DNA reference standards are the factors controlling the precision and reproducibility of flow cytometry data (25).

Since *Thermosynechococcus* E542 is polyploid, successful engineering efforts must integrate the desired mutations into all genome copies to generate stable mutants. Reculturing of a single colony on selective medium needs to be done multiple times until complete segregation of mutations is obtained across all genome copies. The time required to obtain a fully segregated mutant depends on the strain, laboratory conditions, and type of mutation (26). This process requires several weeks, especially in slow-growing cyanobacteria like *Thermosynechococcus* E542, which usually requires more than a week to produce visible colonies on agar plates. Therefore, information about the distribution of genome copy numbers in a population of *Thermosynechococcus* E542 is also essential for constructing mutants. In this way, identifying a condition that produces more mono- or diploid cells could accelerate the transformation process due to faster mutant segregation.

### Variability of the ploidy level of *Thermosynechococcus* E542.

This study has explored the genome copy number variations and their alleged role in *Thermosynechococcus* E542. The results showed that *Thermosynechococcus* E542 tends to maintain ploidy of 3 or 4 under normal growth conditions, nearly the same ploidy in early-growth-phase and stationary-phase cultures, and significantly increased ploidy during the exponential growth phase (Fig. 1C). Therefore, the ploidy level in *Thermosynechococcus* E542 is growth phase regulated. These results are in accordance with those from the model strain Synechococcus elongatus PCC 7942, which also possesses 3 or 4 genome copies per cell in growth and stationary phases (11). Meanwhile, unlike the *Thermosynechococcus* E542 strain, it exhibited increased genome copies (4 to 10 copies per cell) during lag phase (27). It has also been noted that a substantial difference in the ploidy level could not be seen in the model strain *Synechococcus* PCC 7942 and *Thermosynechococcus* E542 during different growth stages. This is in stark contrast to *Synechocystis* sp. PCC 6803, which displayed a massive change in the ploidy level, exhibiting nearly 25 genome copies in low-density cultures and <5 genome copies in higher-density cultures (10). Growth phase-dependent downregulation of the ploidy level is also observed in other bacteria, for example, in Halobacterium salinarum (28). Nevertheless, growth phase-dependent downregulation of the ploidy level is not universal. Some bacteria, e.g., Azotobacter vinelandii, possess nearly 100 genome copies in the stationary phase, while only 4 copies were observed during early exponential phase (29). Knowing the dynamics of ploidy changes across different growth phases is essential for understanding how chromosomes segregate during cell division (30).

The addition of bicarbonates is reported to increase the growth of cyanobacteria (18), including thermophilic cyanobacteria (19). Our previous work tested the effects of 100 mM, 300 mM, and 500 mM bicarbonates and found that 100 mM is the most effective concentration for increasing the growth rate and biomass (6). Hence, 100 mM was chosen for the present study. As ploidy is increased in cultures with higher metabolic activity, a further increase in genome copies was observed with the addition of carbonates (Fig. 1C). A clear shift in the DNA amount can also be seen from the DNA histogram. Meanwhile, the genome copy distribution showed reduced mono-, di-, and triploid cells in these HC cultures (Fig. 3). These results suggest that there is a correlation between increased growth and genome copy number. The direction of the cause and effect of this relationship remains to be determined. Another recent work is in accordance with this hypothesis and suggests that the ploidy level depends on the cellular growth rate of *S. elongatus* (14).

Previous studies proposed that bicarbonate supplementation increases growth by reducing oxidative stress in the unicellular alga Dunaliella salina grown under macronutrient-deficient conditions (31). Although the addition of carbonates to LP cultures does not increase the growth rate, an increased ploidy level (*P* < 0.05 by a *t* test) was found compared to LP conditions, which supports the role of carbonates in managing stress under nutritionally deprived conditions.

The pH of BG-11 after the addition of sodium bicarbonate reaches 9 to 10. Even after a pH adjustment to 8, the growth of cyanobacteria may slightly change the final pH. *Thermosynechococcus* E542 shows good growth at the broader pH range of 6 to 12 (6). Furthermore, no difference in the ploidy level of early-growth-phase cultures was observed in HC cultures, which shows the negligible effect of pH changes during growth.

The availability of phosphates is linked to the ploidy level, based on the DNA structure itself. However, the increase in ploidy after increasing phosphate availability is not as significant as the reduction in the ploidy level after decreasing phosphate availability (10). The growth rate in HP cultures increased after the culture attained an OD above 0.5 compared to the control. The ploidy level also increased, but this increase was <1-fold compared with the control. On the other hand, after phosphate starvation, genome copies decreased from 20 to 2 in Haloferax volcanii (32) and from 27 to 1 in *Synechocystis* sp. PCC 6803 (10). Interestingly, phosphate starvation does not affect average ploidy in *Synechococcus* (30). *Thermosynechococcus* E542 exhibits only minor ploidy changes across different cultivation conditions. In early LP cultures, when phosphates were reduced to 25% of the original concentration, the highest percentage of cells (30%) was observed to contain 5 genome copies, which is higher than the number under any other condition. Similarly, the highest number of cells possessing more than 5 genome copies was also observed in LP cultures. It could be hypothesized that increased genome copies in the early growth phase of low-phosphate cultures and decreased genome copies in the later phase (increased mono- and diploid cells) were the adaptive response of the strain to store the phosphate at first and utilize it in later stages.

In the absence of phosphates, the 5-fold increase in the optical density throughout the experiment was accompanied by a 2-fold decrease in ploidy. At the stationary phase, only 18% of the cells were monoploid. These findings suggest that the E542 strain is likely to maintain the polyploid status even in the absence of a phosphate source. These findings are in accordance with *Synechocystis* sp. PCC 6803 grown under phosphate starvation, in which the reduction in the genome copy number was lower than the increase in the cell number (10). The H. volcanii response to phosphate starvation is different. It shows a high reduction in the genome copy number compared to the increase in cell numbers (32), suggesting the degradation of genomic DNA to liberate phosphate needed for the synthesis of other biomolecules. These findings demonstrate that different polyploid species use different approaches to tackle phosphate starvation. These strategies allow polyploid organisms to adapt their growth in response to phosphate starvation, which is not possible for monoploid species, highlighting one of several evolutionary advantages of polyploidy (15, 33). It is interesting to note that the cell volume and genome copy numbers are suggested to be positively correlated (34–36). In contrast, during phosphate starvation, when the ploidy level was reduced to 50% of the control, microscopic analysis revealed enlarged *Thermosynechococcus* E542 cells (see Fig. S4 in the supplemental material). Another work supports that polyploid bacteria can change chromosomal copy numbers without any change in the gene copy number per unit cell volume (14). Enlarged cells under phosphorus starvation were also observed in Amphidinium carterae, and this deficiency leads to increased contents of carbon, nitrogen, and protein. This appears to be an adaptive strategy to restore growth upon phosphate availability (37). Contents of nitrogen and the carbon reserve polymer cyanophycin increase in phosphate-starved *Synechocystis* sp. PCC 6803 cells (38). A similar mechanism may have contributed to the increased cell size in our strain. Nevertheless, more investigation is required to elucidate this phenomenon.

From these results, it could be deduced that the availability of phosphates and carbonates influences the average ploidy and distribution of genome copy numbers in populations of *Thermosynechococcus* E542 and that by limiting the availability of phosphates, more mono- and diploid cells could be obtained that could facilitate the process for the construction of mutants. The temperature significantly affected ploidy in the mesophilic cyanobacterium *S. elongatus* PCC 7942, and ploidy was inversely correlated with temperature (14). In the current study, the increase of the cultivation temperature from 45°C to 60°C resulted in a shift of the ploidy distribution from triploidy to tetraploidy (Fig. 4). In the course of this temperature shift, the *Thermosynechococcus* E542 cells grew steadily. This could be attributed to the increased expression of heat shock proteins. The increase of RNA polymerase template availability through increased ploidy could be one of the mechanisms of coping with heat stress in thermophilic cyanobacteria. Compared to mesophiles, thermophiles are considered resistant to external fluctuations and typically possess genetic and physiological adaptations to minimize and repair damage caused by multiple types of environmental stresses. These mechanisms usually include the existence of stress-responsive proteins, adaptive stress mitigation mechanisms (39, 40), and compatible solute accumulation (41). All these mechanisms often act in response to various, not only temperature, stresses (41, 42).

*Thermosynechococcus* E542 could grow with 2.5 μg/ml of spectinomycin, while *Synechococcus* PCC 7942 was unable to do so. WT *Thermosynechococcus* E542 showed increased percentages of tetra- and pentaploid cells (Fig. 4). Since spectinomycin inhibits protein translation through binding to the 30S ribosomal subunit, the increased availability of the genomic template and synthesis of rRNA could be one of the mechanisms to cope with the inhibition. In contrast, transformed cells showed that the highest percentage of the population is diploid. This reduced ploidy shows that after the acquisition of the spectinomycin resistance gene, transformed *Thermosynechococcus* could effectively cope with the high antibiotic concentration. As a result, a higher ploidy level was no longer required to compensate for its toxic effect. These results are consistent with the hypothesis that an increase in genome copies acts as backup genetic information to compensate for damage to the other chromosomal copies and helps the cells to cope with extreme conditions (24, 28, 43–46). Although different stress factors altered the distribution of genome copies among the populations on a single-cell basis, *Thermosynechococcus* E542 managed to maintain its average tetraploidy with no significant change compared to the control (*P* > 0.05 by a *t* test). Such robustness in maintaining an average ploidy level suggests the strong defensive and adaptive response of thermophilic cyanobacteria.

### Conclusion.

Three improved ploidy estimation approaches, including qPCR, spectrofluorometry, and flow cytometry, are presented in this work to facilitate the ploidy estimation procedure for cyanobacteria in the future. If the analysis of the heterogeneity of genome copy numbers in a sample is not desired, we found the spectrofluorometric method reasonably sound and fast compared to other methods used in this work. The present study suggests that among the tested conditions, the growth phase and bicarbonate level are the most significant factors impacting the ploidy level of *Thermosynechococcus* E542.

## MATERIALS AND METHODS

### Selected strains and culture conditions.

The cyanobacterium Thermosynechococcus elongatus PKUAC-SCTE542 (here *Thermosynechococcus* E542) (47) was used in this study. The strain was recently deposited in The Freshwater Algae Culture Collection at the Institute of Hydrobiology, Chinese Academy of Sciences (CAS), with the accession number FACHB-2455. A preculture was maintained in conventional BG-11 (48) in a shaking incubator at 45°C with constant agitation (100 rpm) and constant illumination of 70 μmol m^−2^ s^−1^. The stationary-phase preculture was diluted to an optical density at 730 nm (OD_730_) of 0.05 for test conditions. Synechococcus elongatus PCC 7942 was used as a control strain. Its preculture was maintained at 30°C in conventional BG-11. *Thermosynechococcus* E542 cells from different growth stages (lag phase, early growth phase, late growth phase, and stationary phase) were analyzed to test if ploidy is growth phase regulated. The addition of bicarbonate has been shown to increase the growth rate in cyanobacteria (18); hence, bicarbonate (NaHCO_3_) was added to BG-11 to test its effect on growth and ploidy levels. For the determination of genome copy numbers as a function of phosphate availability, various phosphate concentrations (K_2_HPO_4_) were added to BG-11. To determine the effect of temperature on thermophilic cyanobacteria, *Thermosynechococcus* E542 was grown at regular and elevated temperatures (45°C and 60°C), and genome copies per cell were measured. Finally, for the determination of variable ploidy as a function of stress management (14), *Thermosynechococcus* E542 was grown in spectinomycin (1 μg/ml to 20 μg/ml)-containing BG-11, and genome copies were estimated under the most tolerated concentration of spectinomycin. Alternatively, the pETS1 vector carrying a spectinomycin resistance gene was used to transform *Thermosynechococcus* E542 as described previously (6), and ploidy was estimated after growing transformed cells in 50 μg/ml spectinomycin. The OD of the cultures was measured at 730 nm using an Epoch 96-well microplate reader. Cells were collected from lag phase (OD_730_ of <0.1), early growth phase (OD_730_ of 0.2 to 0.3), late growth phase (OD_730_ of 0.8 to 0.9), and stationary phase. *Thermosynechococcus* E542 cells with an initial OD_730_ of 0.05 were cultured in BG-11 without phosphates (no phosphate [NP]) as a control for flow cytometry. After 7 days, the cells (OD_730_ of 0.23) were collected and assigned as diploid after quantitative PCR (qPCR) and spectrofluorometric analysis. NP cells were used as a diploid control. Three biological replicates were used for all tested conditions. Details are provided in Table 1.

**TABLE 1:** Culture conditions used for ploidy determination

Test condition	Culture condition(s)	BG-11		Cell collection			
		K_2_HPO_4_ concn (mM)	NaHCO_3_ concn (mM)	Lag phase (0 h)	Early growth phase (1 h)	Late growth phase (2 h)	Stationary phase (3 h)
Different growth stages	Control	0.23	0	✓	✓	✓	✓

Effect of carbonates	HC	0.23	100	✓	✓

Effect of phosphate	HP	0.92	0	✓	✓
LP	0.0575	0	✓	✓
NP^*a*^	0	0	✓

Effect of phosphate and carbonate	HP+HC	0.92	100	✓	✓
LP+HC	0.0575	100	✓	✓

Elevated temp	60°C	0.23	0	✓

Spectinomycin	WT-spec^*b*^	0.23	0	✓
SP-spec^*c*^	0.23	0	✓

a No-phosphate (NP) cells were used as the diploid control for flow cytometry.

b Wild-type *Thermosynechococcus* E542 cells grown in BG-11 containing 2.5 μg/ml of spectinomycin.

c Transformed *Thermosynechococcus* E542 cells possessing the spectinomycin-resistant pETS1 vector (6) grown in BG-11 containing 50 μg/ml of spectinomycin.

### Cell harvesting, enumeration, and disruption.

Cells collected under each test condition by centrifugation were washed twice with phosphate-buffered saline (PBS) and divided into two portions: one for qPCR or fluorospectrometric analysis and the other for flow cytometry. To analyze ploidy using qPCR and spectrofluorometry, cells were resuspended in distilled water, and an automated cell counter (IC1000; Countstar) was used to count the cells in suspension. Cells were disrupted using 0.75 g of zirconium beads in a bead beater (Shanghaijingxin, China) at 70 Hz for 90 s. The efficiency of cell breakage was verified by microscopy. The resulting suspension was centrifuged, and the supernatant was directly used for qPCR and spectrofluorometry.

### Ploidy determination by quantitative PCR.

Genome copies per cell were calculated using a real-time PCR system (CFX96 real-time system; Bio-Rad) as described previously (10). A fragment of 1,061 bp was amplified from isolated genomic DNA of *Thermosynechococcus* E542 as a template using the PCR conditions described below. It was denoted the “standard fragment” as this purified fragment was used to amplify an “analysis fragment” of 160 bp, internal to the standard fragment. This genome portion is predicted to encode a DUF697 domain-containing protein. Amplification was done with *Pfu* DNA polymerase (Vazyme Biotech, China) at 58°C; primer details are provided in Table 2. The amplified fragment was then gel purified, and the DNA concentration was determined with the Quant-IT dsDNA HS assay kit (Invitrogen) using a spectrofluorometer (RF-5301PC; Shimadzu). Concentrations of DNA molecules were calculated from an online resource (http://cels.uri.edu/gsc/cndna.html). A 10-fold serial dilution of the standard fragment was prepared in parallel with serial dilutions of the cell extract. The 25-μl qPCR mixture consisted of 12.5 μl of TB Green Premix Ex *Taq* II (TaKaRa), 0.8 μl of each primer (10 μM), 2 μl of the template, and 8.9 μl of purified water. An analysis fragment of 160 bp was amplified (58°C) from the dilution series of the standard fragment and the cell extract, the cycle threshold (*C_T_*) values were determined, and a standard curve was plotted. The standard curve was used to calculate the PCR efficiency and to determine genome copies in the test sample. A negative control, without the template, was also performed along with the sample. Details for the primers and fragments are provided in Table 2.

**TABLE 2:** PCR primers and fragments used for qPCR

Fragment	Positions in CP032152^*a*^	Primer sequences (5′–3′)	Product size (bp)	Purpose
Standard	2899–3959	GCAAGGATTTTGTTTGGGGAG	1,061	Amplification of standard fragment from *Thermosynechococcus* E542 genome by conventional PCR
		TCTGGGATTGGGCTATTGGTA		

Analysis	2985–3144	TGTAAACAACTCCCTGCCAAA	160	Amplification of analysis fragment from *Thermosynechococcus* E542 by qPCR
		AAATCTACCACCGCTCCTTTC		

a Positions in the *T. elongatus* genome under ENA accession number CP032152.

### Ploidy determination with a spectrofluorometer.

The supernatant obtained after cell disruption was subjected to spectrofluorometric quantification of DNA using a Quant-IT dsDNA HS assay kit (Invitrogen) according to the manufacturer’s instructions. Briefly, 5 μl of assay reagent was mixed with 995 μl of assay buffer, and 2 to 20 μl of the sample was mixed with 200 μl of the prepared buffer. Readings were taken using a spectrofluorometer (RF-5301PC; Shimadzu) (excitation/emission maxima at ∼502/523 nm). Ten microliters of each of the Quant-iT dsDNA HS standards (2 μg to 100 μg) was also tested in parallel, and a standard curve was plotted between the DNA concentration and the absorbance. Genome copies per cell were deduced from the standard curve using the known cell count in the test sample.

### Ploidy determination by flow cytometry.

Flow cytometry was performed using an Attune NxT flow cytometer as described previously (12). Briefly, ethanol-fixed cells were washed twice with PBS and incubated with RNase A (1 μg/ml) at 37°C for 1 h. Cells were stained with propidium iodide (Sangon Biotech, China) according to the manufacturer’s instructions. No-phosphate (NP) *Thermosynechococcus* E542 cells (OD_730_ of 0.23), identified as diploid cells by qPCR and spectrofluorometry, were used as a control. Analysis of the data was performed with FlowJo v.10, and the numbers of genome copies of individual cells were deduced (12). A paired *t* test and design of experiments (DOE) factor analysis were performed to test the effect of different parameters on the ploidy level.

## Supplementary Material

Supplemental file 1
