# Supplementary material for: The Genome Copy Number of the Thermophilic Cyanobacterium Thermosynechococcus elongatus E542 Is Controlled by Growth Phase and Nutrient Availability
Source: Appl Environ Microbiol. 2021 Apr 13;87(9):e02993-20. doi: 10.1128/AEM.02993-20 (PMC8091003; doi:10.1128/AEM.02993-20)
Supplement: Supplemental file 1 [file AEM.02993-20-s0001.pdf]

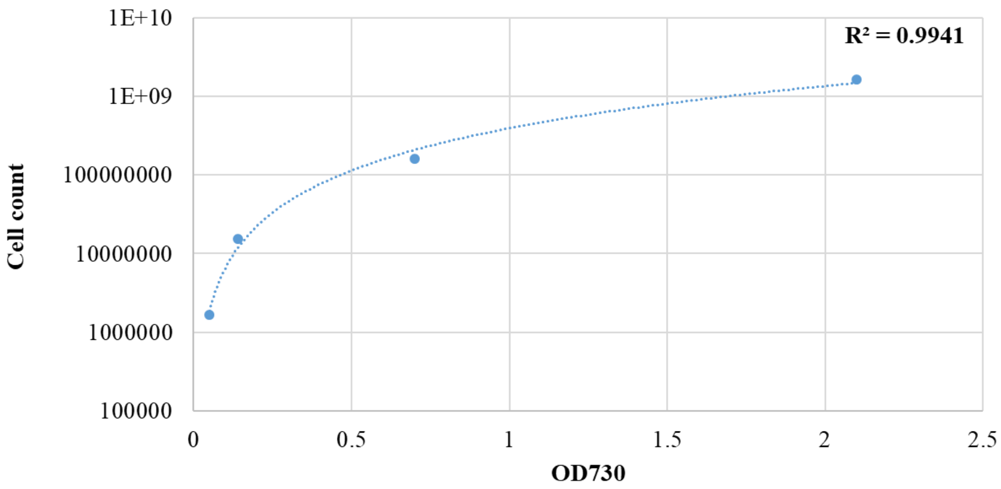

Figure S1. Correlation between optical density (OD) and cell count (Countstar IC1000).

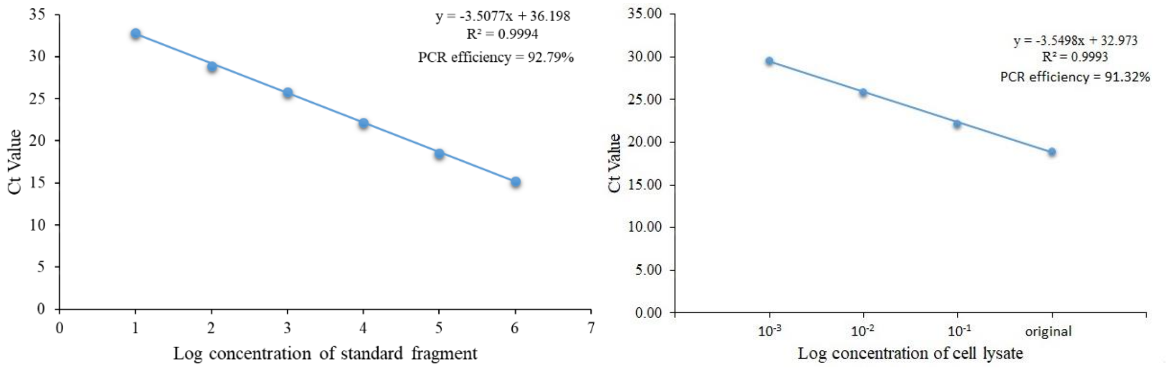

Figure S2. Standard curves constructed with *Thermosynechococcus* E542 standard fragment and cell lysate and deduced PCR efficiency.

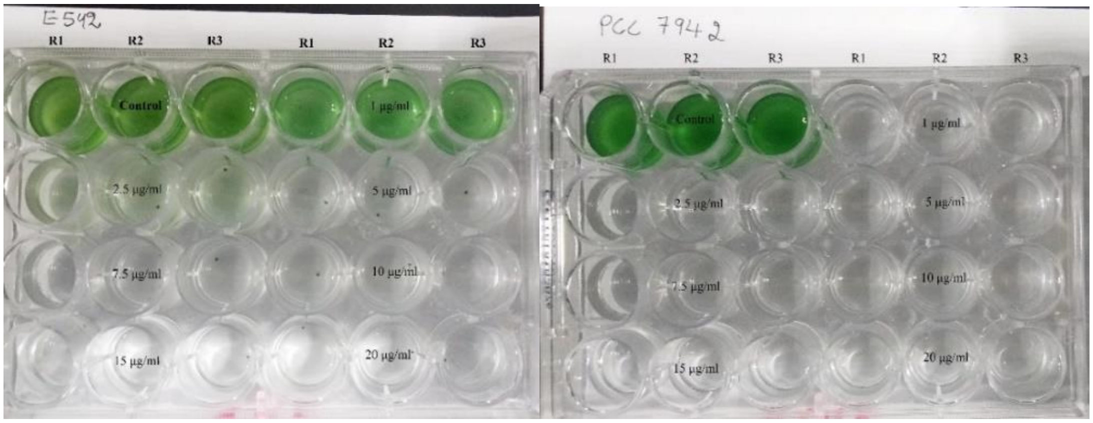

Figure S3. Spectinomycin sensitivity of *Thermosynechococcus* E542 and *Synechococcus elongatus* PCC 7942. Three biological replicates (R1, R2 and R3) were run for each concentration of antibiotic. Row 1: control (Left) and 1 µg/ml spectinomycin (right), Row 2: 2.5 µg/ml spectinomycin (Left) and 5 µg/ml spectinomycin (right), Row 3: 7.5 µg/ml spectinomycin (Left) and 10 µg/ml spectinomycin (right), Row 4: 15 µg/ml spectinomycin (Left) and 20 µg/ml spectinomycin (right).

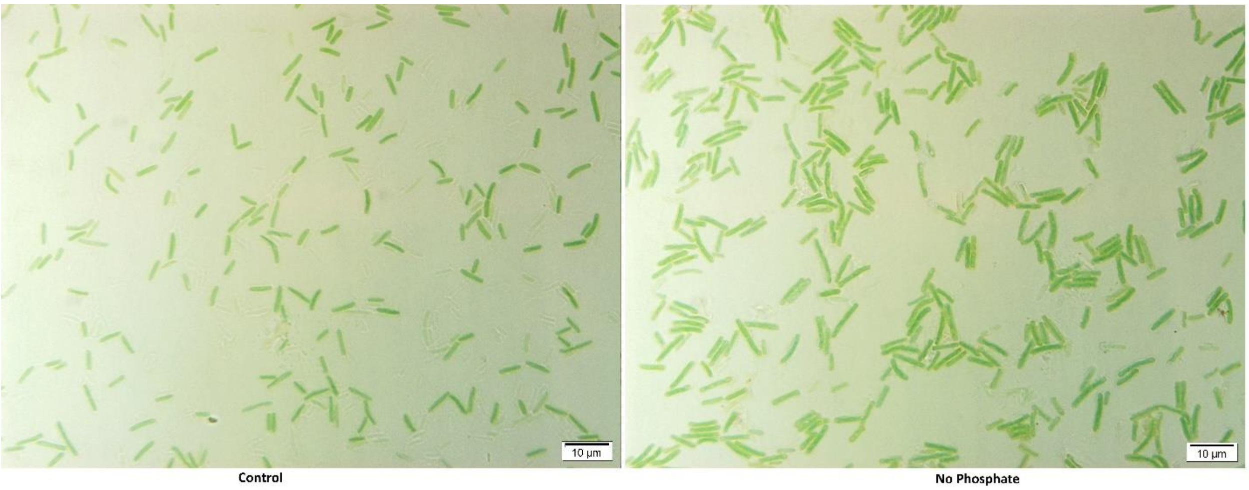

Figure S4. Microscopic analysis of control (left) and no phosphate (right) *Thermosynechococcus* E542. Image is taken at 100X objective lens with immersion oil (Olympus DP72, Japan).

Table S1. Cell lysis efficiency and comparison of qPCR and spectrofluorometer.

Lysis time (seconds)	Genome copies per cell	
	qPCR	Fluorospectrometer
60	5.80 ±0.10	5.20 ±0.06
90	6.20 ±0.20	5.80 ±0.05
120	6.90 ±0.45	6.00 ±0.40

Table S2. Fluorescence intensity readings and their corresponding genome copy numbers. Average fluorescence obtained from no phosphate (NP) *Thermosynechococcus* E542 reference diploid cells was 2721±120 arbitrary unit. The analysis method was adapted from (1).

FI (A.U.)	Actual Genome copy number	Assigned genome copy number
1300-1950	1-1.5	1
1950-3250	1.5-2.5	2
3250-4550	2.5-3.5	3
4550-5850	3.5-4.5	4
5850-7150	4.5-5.5	5
7150-8450	5.5-6.5	6
8450-9750	6.5-7.5	7
9750-11050	7.5-8.5	8
11050-12350	8.5-9.5	9
12350-13650	9.5-10.5	10
13650-14950	10.5-11.5	11
14950-16250	11.5-12.5	12
16250-17550	12.5-13.5	13
17550-18850	13.5-14.5	14
18850+	15+	15+

References

1. Pope MA, Hodge JA, Nixon PJ. 2020. An Improved Natural Transformation Protocol for the Cyanobacterium *Synechocystis sp*. PCC 6803. Front Plant Sci 11:372.
